# Tribological Behavior and Material Removal Mechanisms in Sapphire Lapping Using HFCVD Diamond-Coated Tools

**DOI:** 10.3390/ma19050831

**Published:** 2026-02-24

**Authors:** Wei Feng, Xiaokang Sun, Shuai Zhou

**Affiliations:** School of Mechanical Engineering, Yancheng Institute of Technology, Yancheng 224001, China

**Keywords:** HFCVD, surface morphology, lapping, sapphire wafer, tribological properties

## Abstract

Diamond coatings with three distinct surface textures, namely spherical, pyramidal, and prismatic morphologies, were fabricated using the hot-filament chemical-vapor deposition (HFCVD) method. Scanning electron microscopy (SEM) was employed to analyze the surface morphological characteristics and differences among the coatings. Raman spectroscopic analysis further confirmed that all three diamond films exhibited excellent deposition uniformity and high crystalline quality. A three-dimensional optical microscopy system was used to measure the surface roughness values, which were determined to be Ra 0.423 μm, Ra 0.515 μm, and Ra 0.809 μm, respectively. An HFCVD diamond-coated tool was innovatively employed for the lapping of sapphire wafers, enabling a systematic investigation of the tribological behavior during the lapping process. Based on the experimental results, three morphological material removal models were established. The study demonstrates that the spherical diamond coating achieves a superior surface finish (Ra 0.22 μm) due to its continuous multi-point contact geometry, governed by the agglomerated nanocrystalline structure. Sample 3 had the highest removal rate of 24.3 μm/min. This is related to its surface morphology characteristics and is also due to the two-body contact between the diamond-coated tool and sapphire, offering a high-efficiency alternative for precision machining.

## 1. Introduction

Sapphire (α-alumina, Al_2_O_3_) possesses high hardness and strength, optical transparency in the visible and infrared spectral regions, low thermal conductivity, excellent thermal shock resistance, high wear resistance, a high melting point, outstanding chemical stability, and electrical insulation properties, which enable its widespread application in military electronics, aerospace, and industrial and consumer fields [1,2,3,4]. However, its pronounced brittleness, high hardness, high tensile strength, and chemical inertness render sapphire a typical difficult-to-machine material [5,6]. In general, sapphire wafer fabrication involves initial slicing followed by precision processing techniques, such as lapping and polishing, to obtain the required surface quality. During sapphire lapping, conventional loose abrasive processes suffer from non-uniform abrasive distribution and poorly controlled abrasive motion, which lead to uneven material removal. Fixed abrasive lapping technology has therefore been developed based on traditional lapping methods to address these limitations.

Luo et al. investigated the formulation optimization of mixed soft abrasives composed of SiO_2_ and ZnO, using pure SiO_2_ and pure ZnO abrasives as control groups, and demonstrated the superior balance between processing efficiency and surface quality achieved by the mixed abrasive system [7]. Wang et al. proposed a novel binder-free abrasive particle fabrication method and identified the optimal process parameters through an orthogonal experimental design. When the abrasive particle size was 10 μm, the finishing pressure was 0.15 MPa, and the rotational speed was 150 r/min, high-efficiency and low-damage processing was achieved with a surface roughness Ra not exceeding 0.5 nm and a material removal rate of at least 0.8 μm/h [8]. Uhlmann et al. replaced conventional double-face lapping with double-face grinding using planetary kinematics, which not only shortened processing time and reduced the cost per wafer but also improved wafer quality in terms of flatness, total thickness variation, and surface roughness while extending tool lifetime [9]. Zhang et al. developed a non-corrosive and environmentally friendly green chemical mechanical polishing (CMP) slurry that satisfied the stringent surface quality requirements of high-end sapphire products, with Ra not exceeding 0.15 nm, while simultaneously enhancing the material removal rate and reducing processing costs, thereby providing an alternative to conventional corrosive CMP systems [10]. Xiong et al. developed and evaluated an optimized ceramic-bonded fixed abrasive lapping plate, achieving a material removal rate approximately 30 to 60 percent higher than that of traditional loose abrasive processes, which significantly shortened the lapping cycle [11]. Lee et al. systematically investigated the effects of pressure, velocity, cutting fluid, and wafer type on the self-dressing behavior of fixed abrasive lapping plates to address the critical issue of declining material removal rate caused by abrasive wear [12]. Their results indicated that pressure and velocity had minimal influence on the self-dressing effect, and only the initial material removal rate (MRR) conformed to the Preston equation. Cutting fluids delayed abrasive wear but did not enhance self-dressing, whereas sawn wafers with shape errors of approximately 20 μm in height difference and saw marks generated localized loads that effectively promoted self-dressing, thereby maintaining a high and stable MRR [13].

However, both loose abrasive and fixed abrasive lapping of sapphire suffer from low processing efficiency and the formation of subsurface damage layers to varying degrees. Diamond-coated tools fabricated by chemical vapor deposition (CVD) exhibit high surface hardness, excellent wear resistance, high thermal conductivity, stable chemical properties, and low friction coefficients. These attributes endow diamond-coated tools with long service life, superior machining quality, the capability to accommodate complex substrate geometries, low manufacturing cost, and multi-edge cutting characteristics, rendering them ideal candidates for the efficient machining of hard and brittle non-ferrous materials [14,15,16,17,18]. In this study, the surface texture morphologies of HFCVD diamond coatings are systematically classified and innovatively applied to lapping processes, with the aim of achieving high-efficiency surface machining of sapphire crystals and providing a scientific basis for the precision processing of sapphire and other hard and brittle materials. However, existing studies often overlook the specific impact of surface texture on tribological interactions. This study addresses this gap by systematically classifying morphologies and establishing a direct correlation between crystal habit and the two-body removal mechanism.

## 2. Experiments

**Figure 1:**
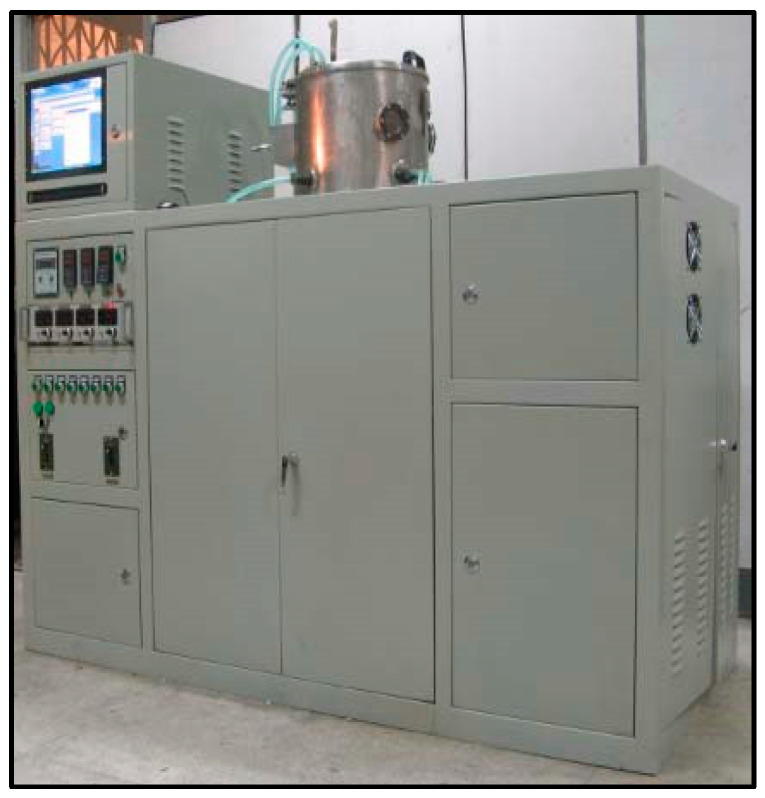
Deposition equipment.

Self-fabricated diamond-coated tools were prepared using the hot-filament chemical-vapor deposition method in a laboratory-scale HFCVD system. Methane and hydrogen were employed as the reactive gas sources, while tungsten filaments with a diameter of 0.4 mm served as the heating elements. The filaments were heated to temperatures ranging from 2000 °C to 2200 °C. A schematic diagram of the deposition apparatus is shown in Figure 1, and the detailed deposition parameters are summarized in Table 1. After deposition, the surface morphology and structural characteristics of the diamond films were examined and analyzed using scanning electron microscopy (Hitachi High-Tech corporation, Tokyo, Japan) and Raman spectroscopy (Horiba Jobin Yvon, Palaiseau, France).

**Figure 2:**
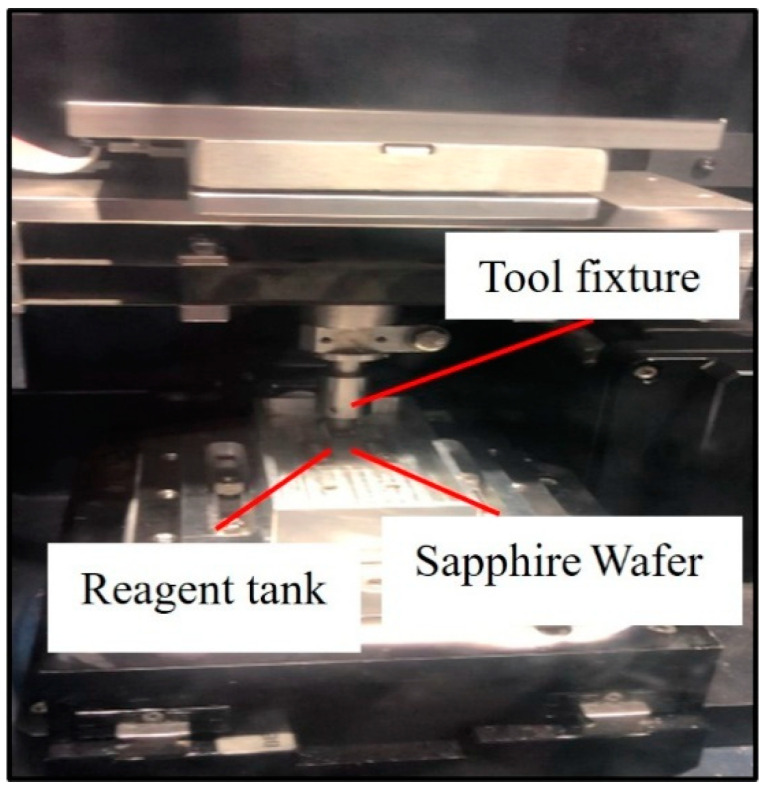
UMT friction and wear testing machine.

To investigate the tribological behavior of sapphire wafer lapping using diamond-coated tools, the as-deposited diamond-coated tools were bonded to a tool holder and paired with sapphire wafers with a diameter of two inches that were fixed on a specimen plate to form a sliding contact pair. The experiments were conducted using a UMT tribometer (Figure 2). Under ambient room temperature conditions of 20 °C–25 °C, lapping tests were performed under water lubrication using CVD diamond-coated tools against sapphire substrates with an applied normal load of 2 N. The evolution of tangential force was monitored for the three CVD diamond coatings with different surface morphologies during the lapping process. After testing, the surface morphology of the diamond coatings was examined and analyzed using scanning electron microscopy and Raman spectroscopy. The surface morphology of the sapphire wafers was characterized using a Keyence optical measurement system and an optical metallurgical microscope.

## 3. Result and Discussion

### 3.1. Surface Characterization of the Deposited Coatings

Diamond films were deposited by hot-filament chemical-vapor deposition in a CH_4_ and H_2_ atmosphere, where the chamber pressure, methane concentration, and deposition temperature play critical roles in determining the film quality and surface morphology. Using the experimental parameters listed in Table 1, three diamond films with distinct surface morphologies were obtained. The surface morphologies of these representative deposited films were characterized by scanning electron microscopy.

**Figure 3:**
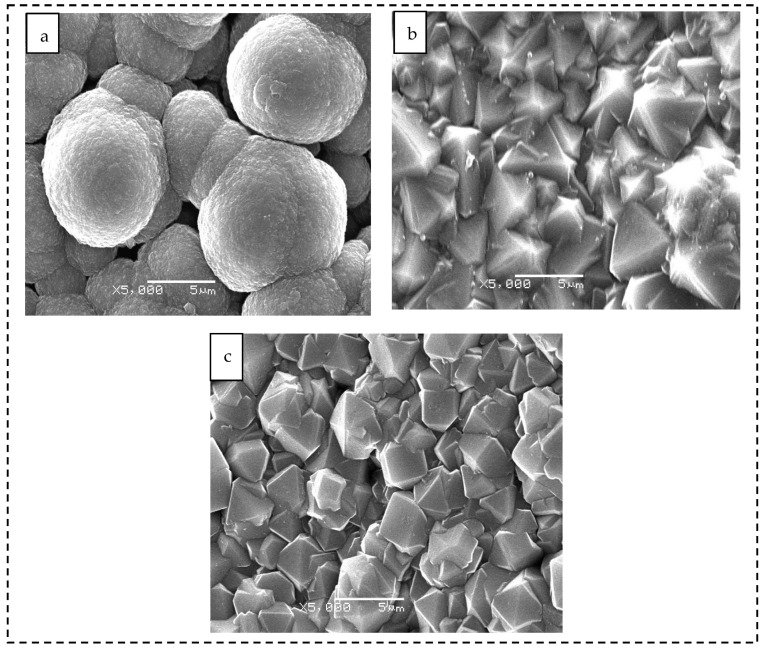
SEM image of the CVD diamond coating tool surface. (**a**) Sample 1, (**b**) sample 2, and (**c**) sample 3.

As shown in the SEM image in Figure 3a, the surface of sample 1 is dominated by globular particles with an overall spherical morphology. The SEM micrographs reveal distinct growth mechanisms. The spherical coating (sample 1) consists of agglomerated nanocrystallites forming clusters of 5–6 μm, induced by the high secondary nucleation rate, which explains the apparent non-uniformity. In contrast, the pyramidal (sample 2) and prismatic (sample 3) coatings exhibit well-faceted micro-crystals with larger, clearer grain boundaries, typical of a lower nucleation density growth mode. As shown in Figure 3b, the surface morphology of sample 2 exhibits a well-defined pyramidal structure, with sharp facets oriented toward the outward viewing direction and a relatively uniform spatial distribution. As shown in Figure 3c, sample 3 presents a morphology in which flat crystal planes are oriented toward the outward viewing direction. The grains display well-defined shapes with distinct grain boundaries.

**Figure 4:**
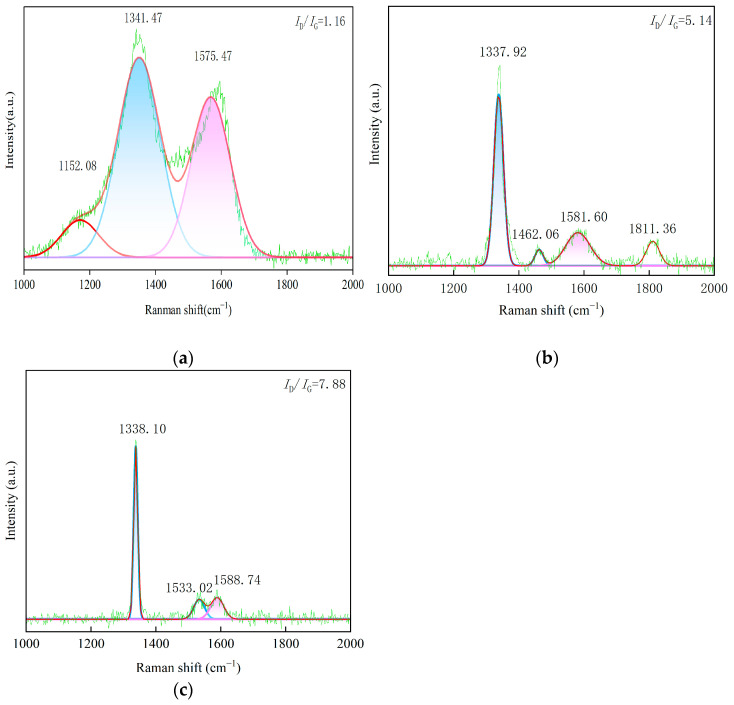
Raman image of the CVD diamond coating tool surface. (**a**) Sample 1, (**b**) sample 2, and (**c**) sample 3.

Prior to analysis, all Raman spectra were baseline-corrected using OriginPro 2024 software to eliminate fluorescence background and allow for accurate comparison. ID/IG is also displayed in the figure. The Raman spectra of the three CVD diamond coatings are presented in Figure 4. In the Raman spectrum of CVD diamond, the characteristic peak of natural diamond appears at approximately 1332 cm^−1^ [19]. The characteristic D band associated with sp^2^ amorphous carbon is located near 1350 cm^−1^. Peaks observed in the range of 1430 cm^−1^ to 1470 cm^−1^ are likely attributed to the trans-polyacetylene modes at grain boundaries. In addition, the characteristic G band of sp^2^ amorphous carbon is observed in the range of 1520 cm^−1^ to 1580 cm^−1^.

The Raman spectral features of the spherical diamond coating are distinctly different from those of sample 2 and sample 3, as shown in Figure 4a. In addition to the diamond characteristic peak located near 1332 cm^−1^, sample 1 exhibits a reduced peak intensity and a relatively broad peak width at this position. A pronounced peak is also observed at approximately 1580 cm^−1^, with a peak intensity lower than that of the 1332 cm^−1^ peak and a comparatively broad linewidth. These spectral characteristics are attributed to the fabrication of sample 1 under conditions that promote a high secondary nucleation rate and the formation of dislocation twins, which inhibit grain growth. As the grain size decreases, the surface grain boundary area increases, facilitating the formation of non-diamond carbon phases at the grain boundaries in polycrystalline diamond films. Consequently, the increased content of non-diamond carbon leads to a broadening of the full width at half maximum of the diamond characteristic peak. In addition, a distinct peak is observed near 1140 cm^−1^ for the spherical diamond coating, which originates from trans-polyacetylene. Because the spherical structures are composed of numerous fine crystallites, smaller grain sizes result in a larger grain boundary area and a higher concentration of trans-polyacetylene at the grain boundaries. There is an inverse relationship between grain size and sp2 content. The nanocrystalline spherical coating (small grains) exhibits a higher volume fraction of grain boundaries, which are the primary sites for sp2-bonded amorphous carbon accumulation.

The Raman spectrum of sample 2 is shown in Figure 4b. The strongest peak appears near 1332 cm^−1^, which is characteristic of the sp^3^ bonding structure of diamond. The Raman intensity around 1330 cm^−1^ is commonly used as an indicator for evaluating the quality of diamond films [20], and under appropriate process parameters, micrometer-scale diamond films typically exhibit a pronounced peak at this position. In contrast, the relative intensities of the D band, G band, and trans-polyacetylene-related peaks are relatively weak, indicating that the sample consists predominantly of micrometer-grained diamond and that the deposited film exhibits high structural quality.

The Raman spectrum of sample 3 is shown in Figure 4c. The most intense peak is located near the diamond characteristic peak at 1332 cm^−1^, indicating that the sample prepared under these conditions consists predominantly of micrometer-grained diamond. The relative intensities of the D band, G band, and trans-polyacetylene-related peaks are comparatively weak, further confirming the high quality of the deposited diamond film.

To provide a rigorous assessment, the peak positions, shifts, and FWHM values were calculated (Table 2). The broader FWHM (full width at half maximum) in sample 1 (12.4 cm^−1^) quantitatively confirms its nanocrystalline nature and higher internal stress state. This morphology evolution aligns with the ‘Synthetic Growth Concept’ [21], where high renucleation rates promote twin formation and modify the bonding state.

**Figure 5:**
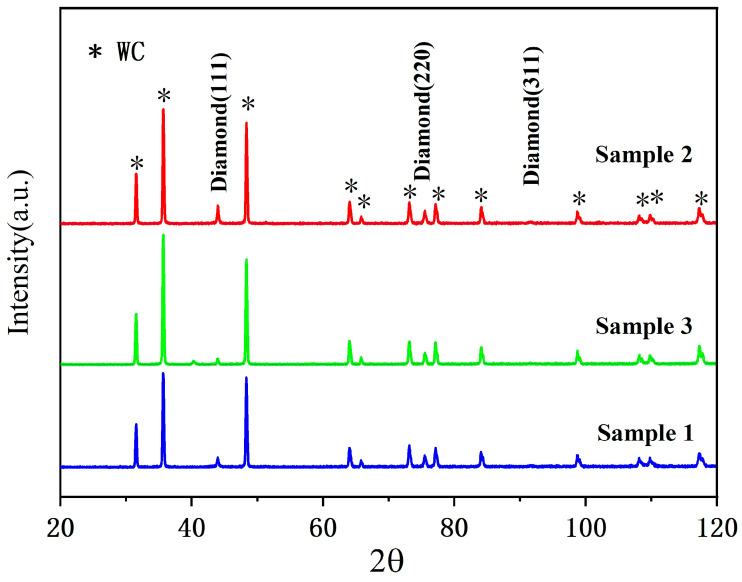
XRD patterns of three morphological diamond thin films.

To further corroborate the structural findings from Raman spectroscopy, X-ray diffraction (XRD) patterns are shown in Figure 5. All samples exhibit characteristic diffraction peaks corresponding to the (111) and (220) planes of cubic diamond, confirming high crystallinity. This structural verification complements the Raman analysis.

**Figure 6:**
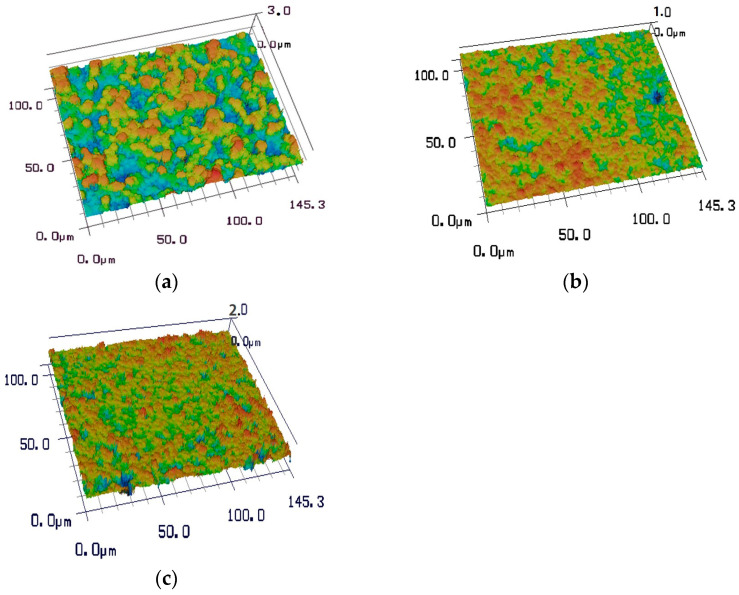
Three-dimensional topography of diamond film surface roughness. (**a**) Sample 1 (Rq 1.239 μm, Ra 0.809 μm), (**b**) sample 2 (Rq 0.524 μm, Ra 0.423 μm), and (**c**) sample 3 (Rq 0.680 μm, Ra 0.515 μm).

The surface roughness of the three diamond films with different morphologies was characterized using a VK-X100 laser scanning confocal microscopy system (Keyence, Neu-Isenburg, Germany), as shown in Figure 6. Sample 2 exhibits surface roughness values of Rq 0.524 μm and Ra 0.423 μm, where Rq represents the root mean square surface roughness and Ra denotes the arithmetic mean surface roughness. Sample 3 shows surface roughness values of Rq 0.680 μm and Ra 0.515 μm, whereas sample 1 presents the highest roughness, with Rq 1.239 μm and Ra 0.809 μm. All samples were deposited on identical substrates and subjected to the same pretreatment procedures, allowing the influence of substrate roughness on the measured diamond film surface roughness to be neglected. Among the three morphologies, the pyramidal diamond film exhibits the lowest roughness. This film grows preferentially along the <111> crystallographic planes, forming inclined pyramidal facets with sharp features that overlap during growth, resulting in indistinct grain boundaries and a reduced surface roughness. The prismatic diamond film, in which flat planes are oriented toward the viewing direction, shows an intermediate roughness. It grows predominantly along the <110> crystallographic planes, leading to uniform vertical growth with clearly defined grain boundaries and pronounced hierarchical features. In contrast, the spherical diamond film exhibits the highest surface roughness. Although this film consists of nanometer-scale diamond grains, the aggregation and clustering of these nanocrystallites during growth result in micrometer-scale spherical agglomerates with relatively large particle sizes.

**Figure 7:**
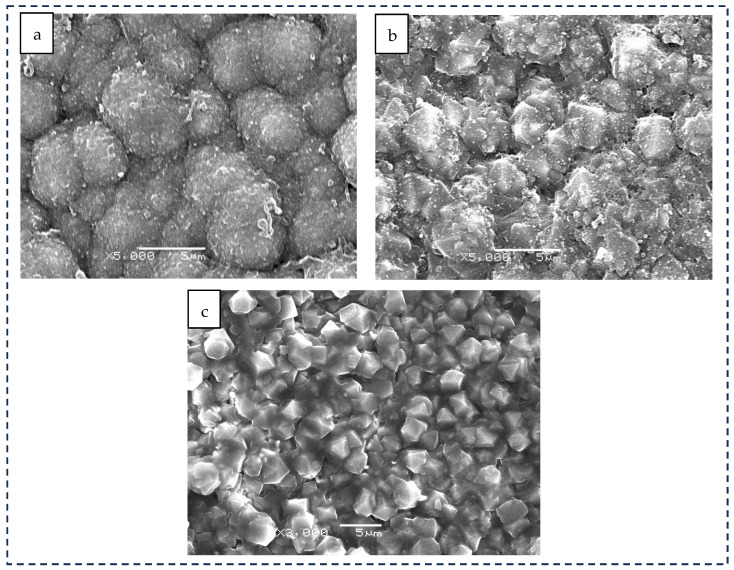
SEM images of CVD diamond coating and single-crystal sapphire after lapping. (**a**) Sample 1, (**b**) sample 2, and (**c**) sample 3.

Figure 7 presents the SEM images of the diamond-coated tools after lapping sapphire substrates. As observed from the images, the original surface structures of the diamond coatings experienced wear during lapping, while the coatings remained well bonded to the substrates, with no evidence of delamination or coating spallation. In Figure 7a, sample 1 exhibits a loss of structural regularity after lapping, and wear debris is observed adhering to the surface. In Figure 7b, wear debris is found accumulated along the grain boundaries of sample 2, and the sharp pyramidal edges are noticeably blunted. In Figure 7c, wear debris is present between the grains of sample 3, and the edges of the cubic or truncated pyramidal grains are either blunted or locally damaged.

**Figure 8:**
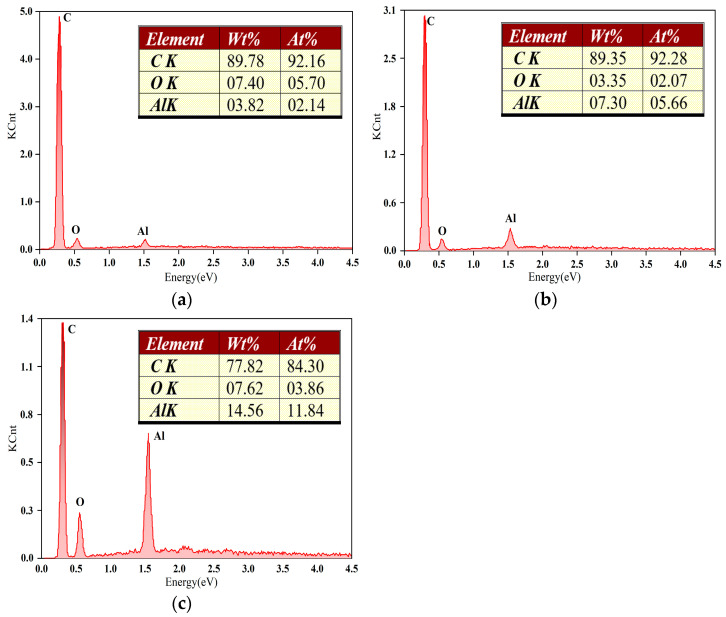
EDS analysis of surface elements of CVD diamond coating after lapping. (**a**) Sample 1, (**b**) sample 2, and (**c**) sample 3.

The elemental distribution on the surface of the CVD diamond coatings after lapping was analyzed using energy dispersive spectroscopy, as shown in Figure 8. The carbon (C) content in the three diamond-coated surfaces is 92.16%, 92.28%, and 84.2%, respectively. The oxygen (O) content is 5.7%, 2.07%, and 3.86%, respectively. The aluminum (Al) content is 2.14%, 5.66%, and 11.84%, respectively. The dominant elements detected are C, O, and Al, indicating that the wear debris is primarily composed of sapphire (Al_2_O_3_). As observed in Figure 8b, the wear debris generated after lapping is predominantly block-like in morphology, whereas the debris produced after lapping with the spherical diamond coating in Figure 8a exhibits a strip-like morphology. This difference suggests that, during sapphire substrate lapping with the spherical diamond coating, sapphire does not undergo purely brittle fracture. Instead, under the embedding, extrusion, and cutting actions of the spherical diamond particles, the material experiences deformation behavior analogous to the lateral plastic flow and accumulation observed in metallic cutting processes. When sapphire is processed under conditions below the critical load for lateral crack initiation, dislocation slip occurs between sapphire grains under shear stress, leading to plastic deformation. Under such conditions, the brittle material can be removed through a plastic flow-dominated material removal mechanism.

**Figure 9:**
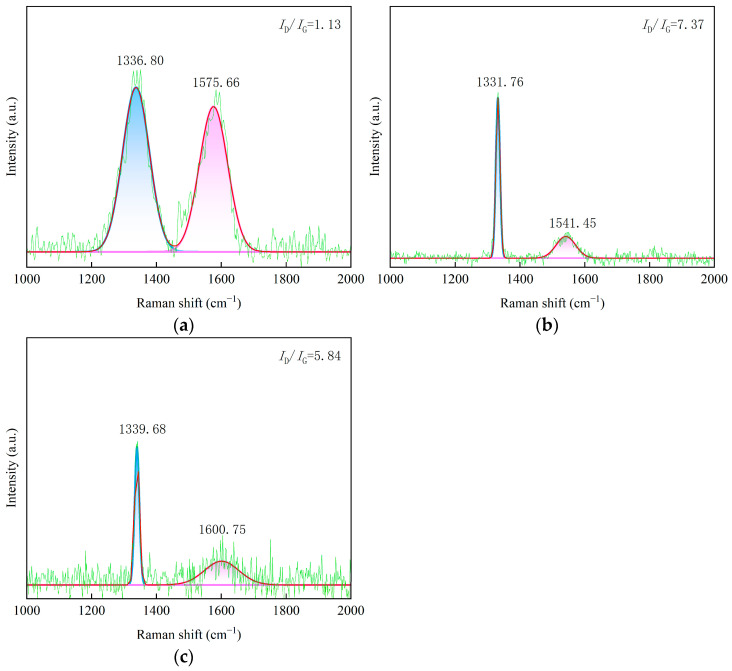
Raman spectra of the CVD diamond coating surface after lapping. (**a**) Sample 1, (**b**) sample 2, and (**c**) sample 3.

Figure 9a,b presents the Raman spectra of sample 1 and sample 2 after lapping. The characteristic Raman peak of natural diamond is located at 1341 cm^−1^. However, owing to the presence of residual stress, the Raman peak position of CVD diamond shifts relative to 1336 cm^−1^. Based on the measured shift in the diamond characteristic peak, the direction and magnitude of residual stress in the diamond coatings can be semi-quantitatively estimated. The residual stress in the CVD diamond films was calculated using the following equation [22,23]:

(1) ρ=k·Δv

(2) $$\Delta v=v_{s}-v_{0}$$

In the equation, *ρ* represents the residual stress in the CVD diamond film, and *k* is a coefficient related to the deposition conditions and substrate material, which is generally taken as a negative value. Δ*v* denotes the relative shift in the Raman peak position, where *v_s_* corresponds to the measured diamond peak position of the sample, and *v*_0_ represents the peak position of natural diamond. When the calculated value of *ρ* is positive, the residual stress is tensile in nature, whereas a negative value of *ρ* indicates compressive residual stress.

Before lapping, the Raman peak of sample 1 was located at 1341 cm^−1^. According to Equation (2), the calculated residual stress in the CVD diamond film was negative, indicating compressive stress. After lapping, the Raman peak shifted to 1338 cm^−1^, suggesting a reduction in the residual compressive stress within the diamond film. Similarly, for sample 2, the Raman peak was located at 1338 cm^−1^ before lapping and shifted to 1331 cm^−1^ after lapping, indicating a decrease in residual compressive stress. This behavior can be attributed to repeated collisions between the coating and the sapphire substrate under applied load during lapping, as well as substantial heat generation resulting from micro-asperity cutting interactions. These effects induce localized strain within the coating, and regions with higher stress concentrations undergo larger strain. As a result, stress redistribution occurs within the coating, leading to stress homogenization and an overall reduction in residual stress.

The full width at half maximum of the characteristic Raman peak of natural diamond is generally approximately 2.3 cm^−1^. The FWHM value of diamond is closely related to the quality of diamond films, including factors such as the crystallinity of diamond grains, the defect density of the film, and the presence of impurities. Therefore, the FWHM of the diamond characteristic peak is regarded as an important parameter for evaluating diamond film quality. As shown in Figure 9c, the FWHM value of sample 3 increases after lapping compared with that before lapping, indicating the formation of sp^2^-bonded graphite, amorphous carbon, and other non-diamond carbon phases at the grain boundaries.

With an increasing content of sp^2^-bonded graphite, amorphous carbon, and other carbon impurity phases in the diamond film, the Raman peak intensity ratio of non-diamond components to the diamond component (*I*_n_/*I*_1332_) decreases accordingly [24]. Compared with the Raman spectra before lapping, the relative intensity ratio of the non-diamond Raman peaks in the range of 1497 cm^−1^ to 1597 cm^−1^ to the diamond peak increases after lapping. This observation indicates that the bonding state of carbon on the surface of the CVD diamond coating changes after lapping, accompanied by an increased content of amorphous carbon at the grain boundaries. The substantial heat generated during lapping can lead to transiently elevated temperatures, which promote the transformation of diamond into non-diamond carbon phases. In contrast, the pronounced increase in the Raman intensity near the diamond peak at 1332 cm^−1^ after testing is attributed to the removal of the growth surface during lapping, resulting in a diamond structure that more closely approaches the maximum structural quality near the substrate interface.

### 3.2. Tribological Characteristics and Lapping Mechanisms of CVD Diamond-Coated Tools

**Figure 10:**
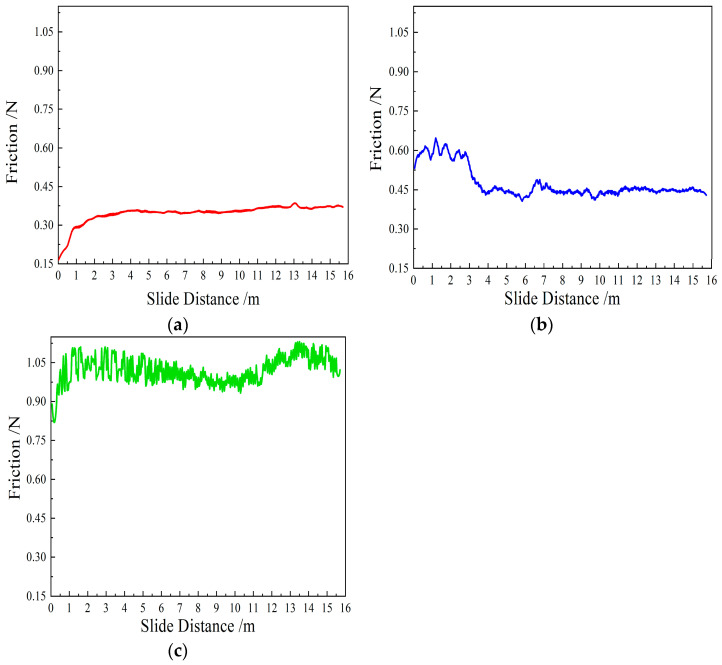
Tangential force variation curve of diamond-coated tool with sliding distance. (**a**) Sample 1, (**b**) sample 2, and (**c**) sample 3.

Figure 10 illustrates the variation in tangential force as a function of sliding distance for the three CVD diamond coatings. As can be seen from the figure, the evolution of tangential force differs markedly among the CVD diamond coatings with different surface morphologies.

As shown in Figure 10a, during the initial stage of lapping, sample 1 forms a very limited real contact area with the uneven sapphire substrate, and most diamond particles within the coating are not subjected to direct normal loading. Owing to the high hardness of CVD diamond, the spherical particles freely abrade the surface asperities of the sapphire substrate, rapidly removing surface irregularities. As a result, the real contact area increases quickly, leading to a gradual rise in tangential force. After reaching a steady-state regime, the tangential force stabilizes at approximately 0.35 N. In comparison with the tangential force evolution of the pyramidal and prismatic diamond coatings, the spherical CVD diamond-coated tool exhibits a lower steady-state tangential force with minimal fluctuation.

As shown in Figure 10b, sample 2 exhibits a pronounced running-in stage followed by a steady-state stage. During the initial stage, the sharp edges of the pyramidal diamond coating penetrate into the sapphire substrate, resulting in a relatively high tangential force. However, the excessive local stress combined with the intrinsically sharp geometry leads to progressive edge blunting with increasing sliding distance. Meanwhile, the pyramidal diamond coating provides a relatively limited chip accommodation capacity, causing the generated wear debris to gradually accumulate within the intergranular spaces and hinder the participation of newly exposed diamond grains in the lapping process. According to tribochemical theories [25], the sharp edges are likely to promote the formation of sp^2^-bonded graphite under high pressure and elevated temperature. Consequently, the tangential force decreases rapidly after the sliding distance reaches a critical value and subsequently stabilizes at approximately 0.45 N.

As shown in Figure 10c, sample 3 exhibits an initial tangential force evolution similar to that of the spherical diamond coating, characterized by a gradual increase from a low value. However, unlike the spherical diamond coating, which reaches a steady state after a sliding distance of approximately 3 m, the prismatic diamond coating attains a stable regime after only about 0.5 m of sliding. This behavior is attributed to the flat rectangular surface morphology of the prismatic diamond coating, which contains edges and corners that enable rapid removal of asperities on the sapphire substrate during the initial stage. As the surface asperities on the sapphire substrate are progressively reduced, the real contact area increases substantially. Owing to its flat rectangular morphology, the prismatic diamond coating provides a larger contact area with the sapphire substrate than the spherical and pyramidal diamond coatings. According to Tabor’s contact friction theory, a larger real contact area results in a higher friction coefficient. Consequently, the prismatic diamond coating exhibits a higher steady-state tangential force, reaching approximately 1.0 N to 1.1 N. During lapping, hard wear debris is generated. Because of the relatively large intergranular spacing of the prismatic diamond coating, part of the debris falls into the intergranular gaps and is removed from the contact interface, while another part acts as abrasive particles that plow between the diamond crystal planes and the sapphire substrate, leading to pronounced fluctuations in tangential force. In addition, within the sliding distance range of 5–9 m, the tangential force gradually decreases, whereas in the range of 9–14 m, the tangential force increases progressively, indicating a certain degree of self-dressing behavior.

**Figure 11:**
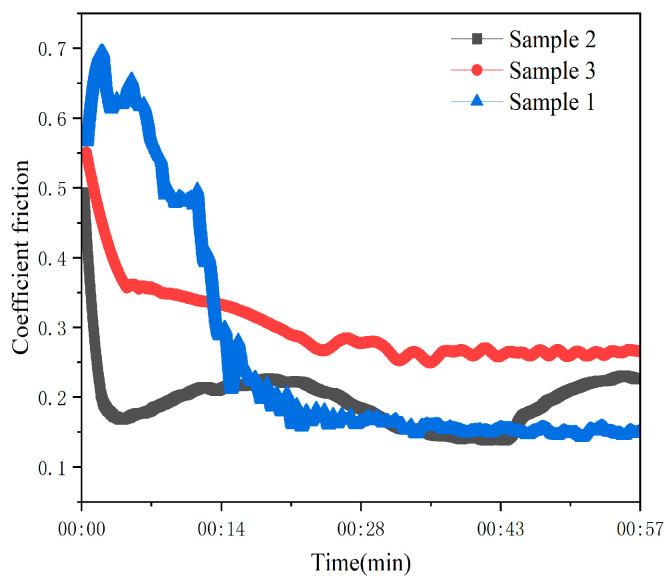
Friction coefficient curve.

Throughout the stable lapping stage, the coefficient of friction (COF) for the spherical tool stabilized at 0.15 as shown in Figure 11, with a low standard deviation, indicating excellent tribological stability compared to the fluctuating forces observed in the prismatic coating.

To study the lapping characteristics of different morphologies of diamond-coated tools on sapphire materials, a single-grain abrasive micromachining model is established. For HFCVD diamond-coated tools lapping sapphire crystals, the grain is embedded in the surface of the sapphire workpiece and is called an effective grain abrasive. The material removal rates of different shapes of diamond-grain abrasives are analyzed. In order to simplify the model, the following assumptions are made before establishing the model:

(1) During the lapping process, the abrasive grain is assumed to be a rigid body and does not undergo elastic deformation under the load.
(2) Diamond grains or particles are closely and uniformly distributed, with consistent size, and do not affect each other.
(3) The lapping pressure is shared by the diamond-coated tool and the workpiece, and the applied external force is borne by the grain abrasive.
(4) The chemical reaction between the coating tool, polishing fluid, and workpiece is not considered.

**Figure 12:**
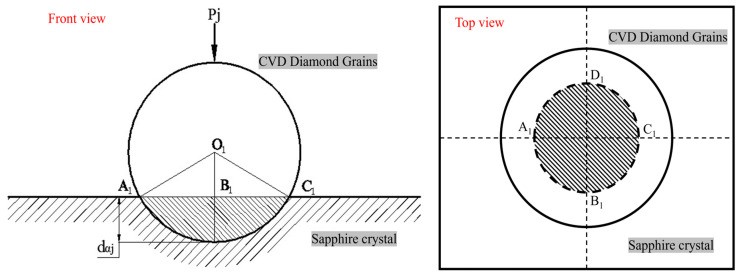
Front-sectional view of spherical CVD diamond (sample 1) particles embedded in sapphire.

Figure 12 schematically shows a biaxial contact diagram of a spherical CVD diamond particle and a sapphire wafer. *D_i_* is the diameter of the diamond particle, *d_αj_* is the depth at which the particle is embedded in sapphire, and *P_j__1_* is the normal force exerted on the grain.

The lapping pressure is evenly borne by the particles in contact with the sapphire workpiece on the coating, so there is

(3) $$N\times P_{j1}=P_{0}\times A_{w}$$

In the formula, *N* is the total number of particles on the coated tool, *P_j_* is the force applied to each particle, *P*_0_ is the pressure acting on the workpiece, and *A_w_* is the surface area of the workpiece being machined. Let *A_j_* represent the area of the pit formed by the abrasive grain indenting into the workpiece surface; then we have

(4) $$A_{j}=\pi \left[{\left(\frac{D}{2}\right)}^{2}-{\left(\frac{D}{2}-d_{\alpha j}\right)}^{2}\right]=\pi \left(Dd_{\alpha j}-\frac{{d_{\alpha j}}^{2}}{4}\right)\approx \pi Dd_{\alpha j}$$

According to the definition of hardness, the normal force *P_j__1_* acting on a single diamond particle is as given in Equation (5) [25,26].

(5) $$P_{j1}=\pi \lambda H_{\alpha }Dd_{\alpha j}$$

In the formula, *H_α_* is the hardness of the sapphire crystal, and λ is a constant representing the influence of factors other than the indenter geometry on hardness, which does not change with the geometry of the indenter. For uniform and dense CVD diamond coatings, it is assumed that the grain abrasive particles are uniformly distributed on the coating surface, and the distance between each grain abrasive particle and its adjacent grains is *l*, so *l* = *D*. When the size of the coated tool is known, the number of grain abrasive particles on a single coated tool can be roughly calculated.

(6) $$N=\frac{ab}{l^{2}}=\frac{ab}{D^{2}}$$

By substituting Equation (4) into Equation (5) and combining with Equation (6), the depth to which a single particle is pressed into the workpiece can be obtained as shown in Equation (7).

(7) $$d_{\alpha j}=\frac{P_{0}A_{w}}{\pi \lambda NH_{\alpha }D}=\frac{P_{0}A_{w}D}{\pi \lambda abH_{\alpha }}$$

**Figure 13:**
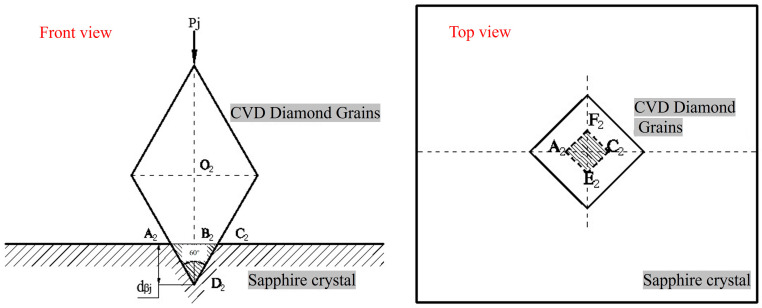
Cross-sectional view of cone-shaped CVD diamond grains (sample 2) embedded in sapphire.

Figure 13 shows a schematic diagram of the two-body contact between a pyramid-shaped CVD diamond grit and a sapphire wafer. For the pyramid-shaped CVD diamond, the entire grit is equivalent to a regular octahedron.

If the abrasive grains of its crystal have an embedding depth of *d_βj_* in the sapphire, then the area *A_j_* of the cross-section A_2_E_2_F_2_C_2_ of the pit formed by the crystal grains indenting the work:

(8) $$A_{j}=\frac{\sqrt{3}}{4}{\left(\sqrt{\frac{3}{2}}d_{\beta j}\right)}^{2}=\frac{3\sqrt{3}}{8}{d_{\beta j}}^{2}$$

The normal force *P_j_*_2_ acting on a single diamond grit particle is as follows:

(9) $$P_{j2}=\frac{3\sqrt{3}}{8}\lambda H_{\alpha }{d_{\beta j}}^{2}$$

The depth to which a single-grain abrasive is pressed into the sapphire workpiece is given by Equation (10):

(10) $$d_{\beta j}=2.15\sqrt{\frac{P_{0}A_{w}}{\lambda abH_{\alpha }}}D$$

**Figure 14:**
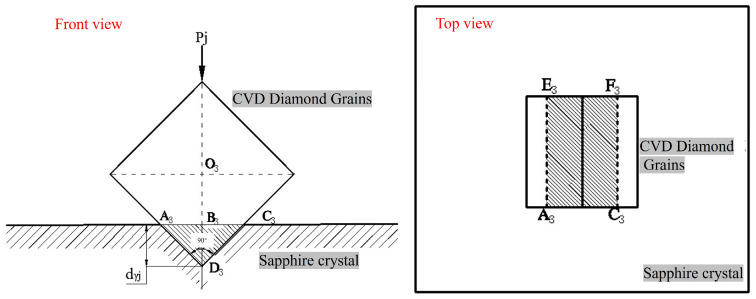
Cross-sectional view of prism-shaped CVD diamond grains (sample 3) embedded in sapphire.

Figure 14 is a schematic diagram of the two-body contact between prism-shaped CVD diamond grits and a sapphire wafer. For the prism-shaped diamond grits, the pit formed on the workpiece surface by the indentation of the grit is a rectangle A_3_C_3_F_3_E_3._

The lapping grains of its crystal have an embedding depth of *d_γj_* in sapphire, and the area *A_j_* of its cross-section A_3_C_3_F_3_E_3_ is:

(11) $$A_{j}=2Dd_{\gamma j}$$

The normal force *P_j_*_3_ acting on a single diamond grit particle is as follows:

(12) $$P_{j3}=2\lambda H_{\alpha }Dd_{\gamma j}$$

(13) $$d_{\gamma j}=\frac{P_{0}A_{w}}{2\lambda NH_{\alpha }D}=\frac{P_{0}A_{w}D}{2\lambda abH_{\alpha }}$$

The depth to which a single-grain abrasive is pressed into the sapphire workpiece is as given in Formula (13).

It can be seen from Equations (10)–(13) that for CVD diamond-coated tools, the depth of penetration of a single-grain abrasive *d_γj_* is not only related to the normal force on the diamond-grain abrasive and the material of the workpiece but also to the shape of the grain abrasive, the size of the grain abrasive, and the diameter of different CVD diamonds. For the same diamond-grain abrasive, when conditions remain unchanged, the larger the equivalent diameter D of the CVD diamond-grain abrasive, the greater the depth of penetration *d_j_* into sapphire. It can be seen from Equations (7), (10), and (13), that the penetration depth of prismatic CVD diamond > than that of the penetration depth of pyramidal > and that of spherical CVD diamond under the same effective particle size and pressure. This is also the reason for the difference in the tangential force of the three different types of diamond-coated tools, as shown in Figure 10. The specific depth can be calculated through the model, which provides a theoretical model basis for further in-depth study of the mechanism.

Substituting the experimental parameters (normal load = 2 N, sapphire hardness ≈ 20 GPa) into Equation (13), the theoretical penetration depth for the prismatic coating is calculated to be 0.85 μm. This value is consistent with the measured surface roughness (Ra 0.809 μm), validating the proposed geometrical model.

**Figure 15:**
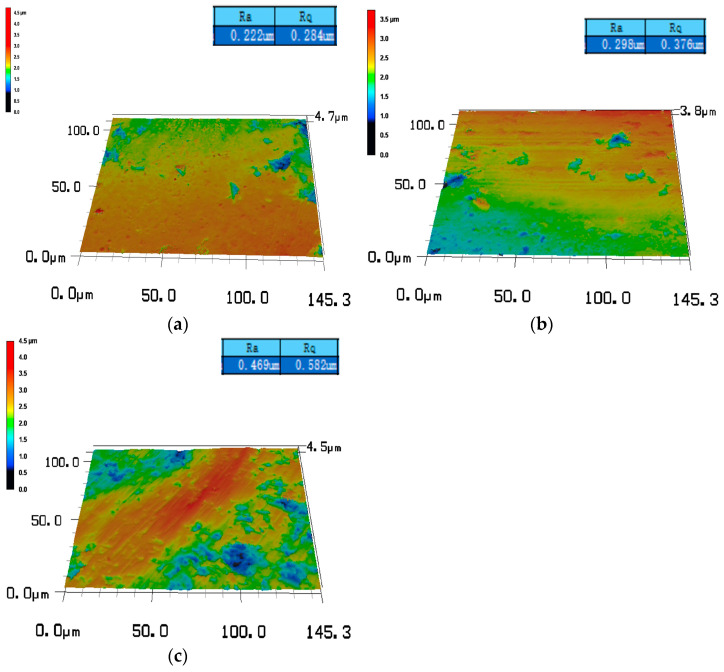
Three-dimensional surface morphology. (**a**) Sample 1, (**b**) sample 2, and (**c**) sample 3.

**Figure 16:**
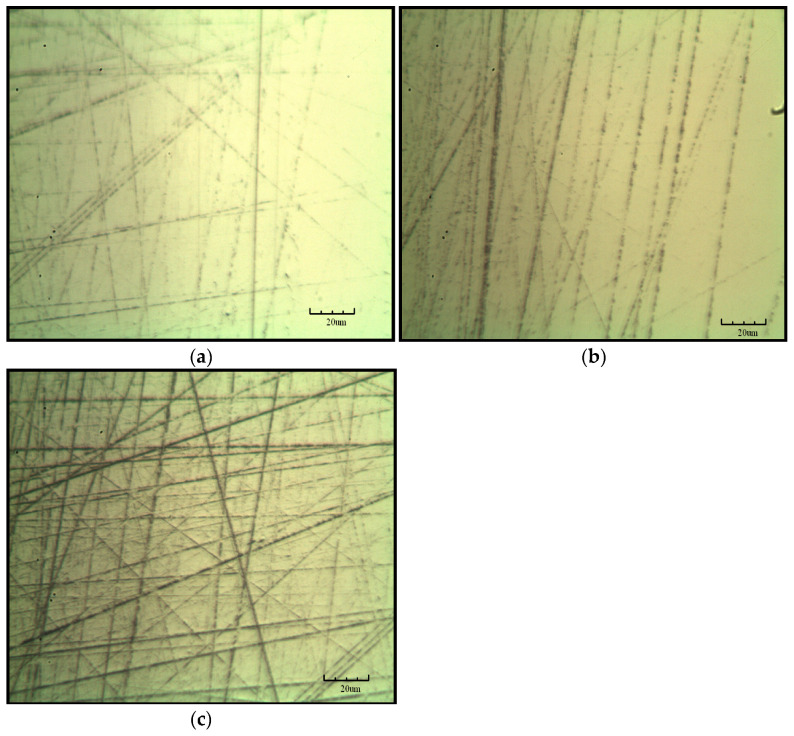
Metallographic microscope image. (**a**) Sample 1, (**b**) sample 2, and (**c**) sample 3.

Figure 15 presents the surface roughness map of the sapphire spherical surface after lapping using the spherical diamond-coated tool, obtained by a Keyence optical microscopy system. The surface roughness Ra values are 0.22 μm, 0.299 μm, and 0.469 um representing a substantial improvement in surface quality compared with that achieved by milling and grinding crown-forming processes and indicating that a favorable lapping performance has been preliminarily attained. However, further magnification reveals that a certain degree of disordered scratches is still present on the processed surface, as shown in the optical metallographic micrograph in Figure 16. From a microscopic perspective, these scratches are primarily attributed to compressive and sliding interactions between diamond crystallites and the sapphire surface during lapping, which lead to localized abrasive scratching. Therefore, further processing of the sapphire surface is still required subsequently.

**Figure 17:**
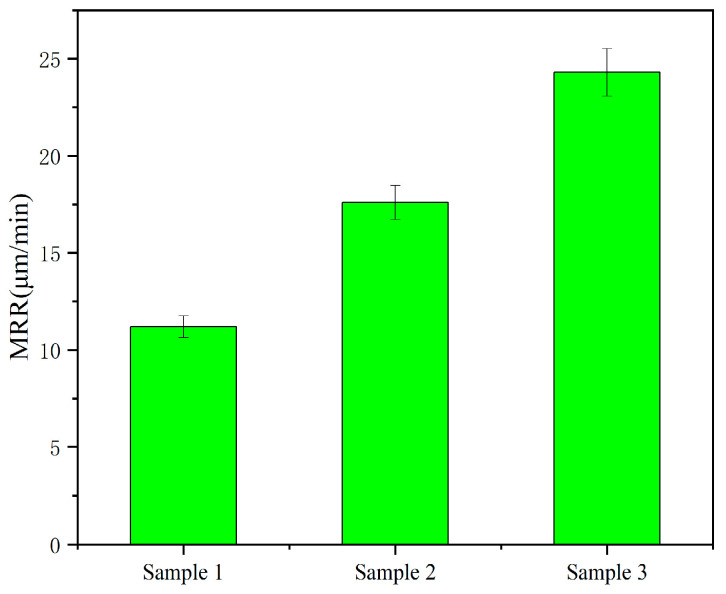
MMR of three diamond films.

Figure 17 is a bar chart illustrating the material removal rate (MRR) of sapphire when three types of diamond coatings are paired with diamond for friction grinding. It can be observed that sample 3 (prismatic shape) exhibits the highest removal rate, sample 2 (pyramidal shape) shows a medium removal rate, and sample 1 (spherical shape) has the lowest removal rate, with the corresponding values being 11.2 μm/min, 17.6 μm/min, and 24.3 μm/min, respectively. This result is consistent with the single abrasive grain removal model established earlier. Under the same pressure, the prismatic shape achieves the maximum indentation depth, thereby yielding the highest removal rate, which is directly associated with its surface morphology. This further verifies the correctness of the established model, and the model can be applied to the machining of similar materials. Consequently, it is concluded that diamond-coated tools can serve as efficient grinding tools for sapphire materials, and the machining quality can be controlled by regulating the surface morphology of the coating.

The reason for the efficient machining is that the workpiece and tool are in two-body contact, rather than three-body contact with free abrasives. The traditional free abrasive grinding is a three-body contact process. During machining, the abrasive slurry flows between the workpiece and the processing tool (or substrate), with the abrasive serving as an intermediate medium to form a “tool-abrasive-workpiece” ternary contact system. The abrasive exerts a grinding effect on the workpiece surface through rolling, sliding, impact, and other means [27].

Our innovative method adopts a two-body contact mode. The HFCVD diamond coating fixes the abrasive on the substrate surface, avoiding the problems of disordered distribution, mutual collision and wear, and loss of abrasives in the loose abrasive slurry. Each diamond particle on the coating surface can participate in the machining process efficiently, and the abrasive utilization rate is increased from less than 30% in the three-body contact to more than 80% [28]. At the same time, the cutting angle and contact pressure of the fixed abrasive are easier to control. By optimizing the coating thickness, grain size, and surface morphology, the uniform distribution of cutting load can be achieved, which greatly reduces the energy loss during the machining process and improves the machining efficiency compared with the three-body contact mode. The contact position and contact load between the abrasive and the workpiece have clear controllability, which avoids the uneven cutting depth caused by the random rolling and sliding of loose abrasives in the three-body contact. In addition, the micro-slip phenomenon at the contact interface can be effectively suppressed by optimizing the structure of the fixed abrasive. Furthermore, the HFCVD diamond coating has extremely high hardness, wear resistance, and surface flatness; it is not susceptible to abrasion, shedding, or chipping during cutting and can effectively reduce scratches, microcracks, and surface roughness fluctuations on the workpiece surface, enabling stable acquisition of higher machining accuracy. It eliminates the processes of preparation, circulation, and recovery of abrasive slurry; reduces environmental pollution caused by abrasive waste and slurry leakage; lowers the process cost and environmental governance pressure; and conforms to the development trend of green machining.

## 4. Conclusions

Three distinct and readily distinguishable CVD diamond coatings with representative surface morphologies were fabricated by hot-filament chemical-vapor deposition. The spherical CVD diamond coating (sample 1) corresponds to a nanocrystalline diamond film, in which spherical agglomerates are composed of numerous fine crystallites. The pyramidal CVD diamond coating (sample 2) exhibits a pronounced textured morphology, characterized by protruding sharp edges and pyramidal grains with apexes oriented toward the outward viewing direction. The prismatic CVD diamond coating (sample 3) also shows strong texturing, with flat crystal planes oriented toward the outward viewing direction, well-defined grain morphologies, and distinct grain boundaries.

The three CVD diamond coatings with different surface morphologies have significantly different tangential force evolutions: the spherical coating has relatively low tangential force with minimal fluctuation, the pyramidal coating’s tangential force decreases from an initially high value to a lower steady level, and the prismatic coating’s tangential force first decreases, then increases, showing a certain degree of self-dressing behavior. A single-grain abrasive micro-removal model is established. Under the same effective particle size and pressure conditions, the penetration depth is as follows: prismatic CVD diamond > pyramidal CVD diamond > spherical CVD diamond.

After processing sapphire wafers with three types of diamond film tools, the surface roughness values of the wafers are Ra 0.22 μm, 0.299 μm, and 0.469 μm, respectively. The material removal rates are 11.2 μm/min, 17.6 μm/min, and 24.3 μm/min, respectively. The removal mechanism between the diamond coating and the sapphire is two-body contact. Achieving uniform distribution of cutting load can significantly reduce energy consumption during the machining process, making it a new, efficient machining method.

## Figures and Tables

**Table 1:** Growth parameters of CVD diamond coating.

Sample	Pressure (kPa)	Substrate Temperature (°C)	Methane Concentration%	Total Gas Flow Rate (SCCM)
1	1	780	3	300
2	3.3	780	1.5	300
3	2.5	800	1	300

**Table 2:** Raman peak assessment.

Sample	Peak (cm^−1^)	Shift	FWHM (cm^−1^)	Quality
Sample 1 (Spherical)	1330.5	−1.5	12.4	Nanocrystalline
Sample 2 (Pyramidal)	1332.2	+0.2	4.5	High Quality
Sample 3 (Prismatic)	1332.1	+0.1	5.1	High Quality

## Data Availability

The original contributions presented in this study are included in the article. Further inquiries can be directed to the corresponding author.
